# Metabolic Engineering of *Komagataella phaffii* for Xylose Utilization from Cellulosic Biomass

**DOI:** 10.3390/molecules29235695

**Published:** 2024-12-02

**Authors:** Jongbeom Park, Sujeong Park, Grace Evelina, Sunghee Kim, Yong-Su Jin, Won-Jae Chi, In Jung Kim, Soo Rin Kim

**Affiliations:** 1 School of Food Science and Biotechnology, Kyungpook National University, Daegu 41566, Republic of Korea; bum5743@gmail.com (J.P.); tya198@naver.com (S.P.); graceevelina@gmail.com (G.E.); 2 Research Institute of Tailored Food Technology, Kyungpook National University, Daegu 41566, Republic of Korea; sunghee.kimmmm@gmail.com; 3 Department of Food Science and Human Nutrition, University of Illinois at Urbana-Champaign, Urbana, IL 61801, USA; ysjin@illinois.edu; 4 Species Diversity Research Division, National Institute of Biological Resources, Incheon 22689, Republic of Korea; wjchi76@korea.kr; 5 Department of Food Science & Technology, Institute of Agriculture and Life Science, Gyeongsang National University, Jinju 52825, Republic of Korea

**Keywords:** *Komagataella phaffii*, xylose metabolism, promoter library, lignocellulose, kenaf

## Abstract

Cellulosic biomass hydrolysates are rich in glucose and xylose, but most microorganisms, including *Komagataella phaffii*, are unable to utilize xylose effectively. To address this limitation, we engineered a *K. phaffii* strain optimized for xylose metabolism through the xylose oxidoreductase pathway and promoter optimization. A promoter library with varying strengths was used to fine-tune the expression levels of the *XYL1*, *XYL2*, and *XYL3* genes, resulting in a strain with a strong promoter for *XYL2* and weaker promoters for *XYL1* and *XYL3*. This engineered strain exhibited superior growth, achieving 14 g cells/L and a maximal growth rate of 0.4 g cells/L-h in kenaf hydrolysate, outperforming a native strain by 17%. This study is the first to report the introduction of the xylose oxidoreductase pathway into *K. phaffii*, demonstrating its potential as an industrial platform for producing yeast protein and other products from cellulosic biomass.

## 1. Introduction

Cellulosic biomass can serve as an excellent source of biofuels and biochemicals, addressing global issues such as environmental pollution and energy depletion caused by today’s petroleum-based economy [1,2]. It is widely available in nature and is an inexpensive resource that can avoid food conflict [3,4]; hence, it is considered an attractive biomass that sustainably meets the industrially viable metrics. Cellulosic biomass consists of three major biopolymer components, namely cellulose, hemicellulose, and lignin [5], of which cellulose and hemicellulose are primarily composed of glucose and xylose, respectively. These abundant sugars can be converted into diverse products through microbial fermentation [6]. Kenaf stands out as a promising cellulosic biomass due to several advantages. It exhibits rapid growth and demands minimal resources and energy for cultivation and processing [7]. Additionally, valuable sugars can be easily obtained through simple pretreatment. Utilizing the liquid fraction obtained after hydrolysate centrifugation as a culture substrate proves advantageous, as it enables the production of high-concentration cellular products without the need for complex and expensive purification processes to remove solid biomass residues. Recent studies have demonstrated that kenaf hydrolysates can be effectively fermented to produce bioethanol and other valuable biochemical, such as organic acids and xylitol, using engineered microbial strains [8,9,10,11].

The methylotrophic *Komagataella phaffii* (formerly known as *Pichia pastoris*) stands out among yeasts as an excellent production host for single-cell and recombinant proteins. This industrial strain has a rapid growth rate, utilizes a well-established methanol-inducible system, and enables easy purification of substances, allowing for the high-concentration production of proteins [12,13]. Furthermore, yeast-derived single-cell protein is nutritionally rich, containing high contents of micronutrients (i.e., vitamins and essential amino acids), along with nucleic acids applicable as flavors. The generally recognized as safe (GRAS) *K. phaffii* receives extensive research attention as a yeast protein source to substitute animal-derived proteins for food and feed applications [14,15], attributed to the significantly lower carbon content and spatial constraint of microbial proteins compared to animal proteins. 

Moreover, the well-established methanol expression system in *K. phaffii* provides advantages in producing significant quantities of industrially valuable recombinant enzymes, such as lipase and protease [13,16]. However, relying on methanol as the sole carbon source has been often economically and technically unattractive due to the high fluctuation in crude oil prices, the source of methanol [17,18], and methanol-induced cytotoxicity [19,20]. To address this issue, the growth performance of *K. phaffii* can be enhanced by supplying a mixture of methanol and glucose [21]. Therefore, cellulosic biomass can serve as an alternative or complementary carbon source (i.e., glucose and xylose) for various biotechnological applications in *K. phaffii*, including the production of industrial enzymes, fuels, and chemicals. Moreover, glucose, the major sugar constituent of cellulosic biomass, can be natively used as the carbon source in *K. phaffii* [22]. Due to its ready availability, cost-effectiveness, and easy metabolism by *K. phaffii*, glucose is commonly used as the sole carbon source or mixed feed with methanol in media, supporting cellular growth and facilitating the expression of recombinant proteins [21,23].

Unfortunately, xylose, one of the most abundant sugars in cellulosic biomass, cannot be fermented by most microorganisms [24]. *K. phaffii* is either unable to utilize xylose as the sole carbon source or exhibits a plodding metabolism [25,26]; this limited xylose-assimilation ability results in a low yield of the target product. Therefore, numerous attempts have been made to introduce the heterologous xylose metabolic pathway into industrial microbial hosts lacking this capability [27,28,29]. In bacteria, strains were developed to metabolize xylose mostly through the introduction of the xylose isomerization pathway, involving xylose isomerase (XI) and xylulokinase (XK) [21,27,28]. Meanwhile, in yeasts, the xylose oxidoreductase pathway, using xylose reductase (XR), xylitol dehydrogenase (XDH), and XK, was introduced [29,30,31].

Improving xylose metabolism in *K. phaffii* increases the substrate efficiency of cellulosic biomass, contributing to cost reduction and environmentally friendly production. While some researches have engineered *K. phaffii* for improved xylose utilization through the heterologous expression of the XI–XK pathway [26] and adaptive laboratory evolution [32], additional strategies are still needed to attain the commercial viability. Introducing the xylose oxidoreductase pathway into *K. phaffii* is a strategy to enhance xylose metabolism. In certain studies, the heterologous expression of the XR-XDH pathway in other yeasts has shown a faster rate of xylose metabolism compared to the xylose isomerase pathway, resulting in higher growth and xylose consumption [33,34]. However, addressing the occurrence of cofactor imbalance and the accumulation of xylitol as a byproduct can be achieved by regulating the expression of genes related to this metabolism.

In heterologous reactions that involve different enzymes, optimizing the gene expression ratio emerges as a useful method to increase sugar consumption and the yield of the target product [35]. Through fine-tuning of gene expression, bottlenecks associated with imbalance between growth and products (i.e., recombinant protein production) [36,37] and the accumulation of intermediate metabolites [38] can be effectively addressed.

In this study, a heterologous, oxidoreductive xylose-metabolic pathway consisting of the *XYL1*, *XYL2*, and *XYL3* genes was introduced into *K. phaffii*. Using a promoter library with varying strengths, the expression levels of these three genes were optimized. The optimized strain was then evaluated for xylose fermentation in cellulosic biomass hydrolysates.

## 2. Results

### 2.1. Expression of a Heterologous Xylose-Metabolic Pathway in Komagataella phaffii Under the Control of Strong Promoters

In previous studies, the heterologous expression of a xylose-metabolic pathway comprising the *XYL1*, *XYL2*, and *XYL3* genes from *Scheffersomyces stipitis* has been successfully demonstrated in *S. cerevisiae* [38,39,40]. To develop a xylose-fermenting *K. phaffii* strain, these genes were introduced under the control of strong, constitutive promoters native to *K. phaffii*: *GAPDHp*, *ENO1p*, and *PET9p*, respectively. Despite some known shared promoters between *K. phaffii* and *S. cerevisiae*, promoters from *S. cerevisiae* were ineffective in *K. phaffii* (Supplementary Figure S1) [41]. In this study, we utilized native promoters of *K. phaffii*, whose strengths have been confirmed in previous research [42].

**Figure 1:**
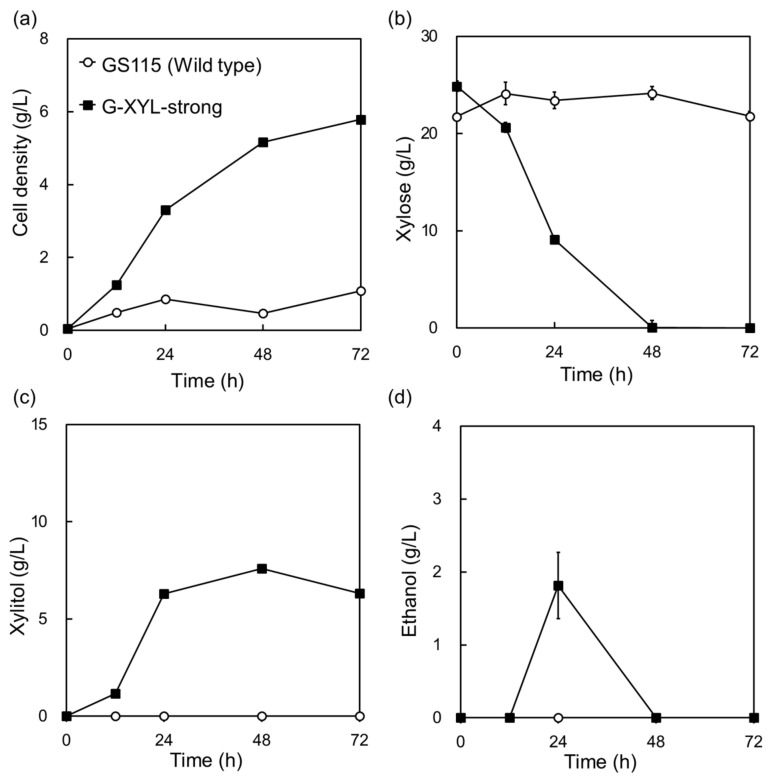
Expression of a heterologous xylose-metabolic pathway in *Komagataella phaffii* regulated by strong promoters, leading to the generation of the G-XYL-strong strain. (**a**) Cell density, (**b**) xylose consumption, (**c**) xylitol production, and (**d**) ethanol production were measured in YP medium containing 20 g/L xylose, at 30 °C and 130 rpm. The pathway genes (*XYL1*, *XYL2*, and *XYL3*) were derived from native xylose-fermenting yeast *Scheffersomyces stipitis*. These genes were expressed under the control of *K. phaffii* promoters, including *GAPDHp*, *ENO1p*, and *PET9p*. Data represent the mean ± standard deviation of three independent biological replicate, with error bars reflecting the observed variability.

The engineered *K. phaffii* strain (G-XYL-strong) was able to grow on xylose as the sole carbon source, whereas the native strain showed no growth. The engineered strain completely consumed 20 g/L xylose within 48 h, producing 2 g/L ethanol (Figure 1). However, a significant portion of the consumed xylose was converted to and accumulated as xylitol, indicating that the heterologous xylose-metabolic pathway requires further optimization.

### 2.2. Optimization of a Heterologous Xylose-Metabolic Pathway in Komagataella phaffii Using a Promotor Library

**Figure 2:**
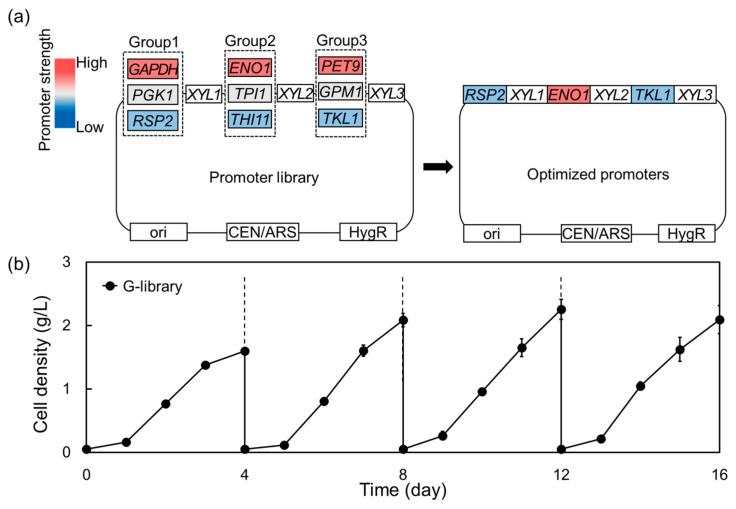
Optimization of a heterologous xylose-metabolic pathway in *Komagataella phaffii* using a promoter library. (**a**) Construction of promoter library by a random DNA assembly, and the optimized promoters identified from a best-growing isolate. (**b**) Enrichment of yeast library (*K. phaffii* GS115 with promoter library) on xylose by four consecutive sub-cultures, after which colonies were isolated for growth evaluation. Enrichment was performed in YP medium containing 200 g/L xylose supplemented with 200 μg/mL hygromycin B at 30 °C and 130 rpm.

For optimizing the expression levels, three constitutive promoters with different strengths were paired with each xylose-metabolic gene, *XYL1*, *XYL2*, and *XYL3*, resulting in 27 possible combinations in the promoter library (Figure 2a). *K. phaffii* GS115 transformants with the promoter library plasmids were serially sub-cultured in xylose to enrich better-growing transformants (Figure 2b). Among four colonies isolated from enriched culture, two (S9 and S17) showed higher growth on xylose compared to the G-XYL-strong strain (Supplementary Figure S2). Both of the better-growing isolates were confirmed to have a strong promoter (*ENO1p*) regulating *XYL2* expression and relatively weak promoters (*RSP2p* and *TKL1p*) regulating *XYL1* and *XYL3*, respectively (Supplementary Table S1).

This result indicates that a high expression of *XYL2* along, with a lower expression of *XYL1* and *XYL*3, is the critical consideration for efficient xylose metabolism in *K. phaffii*, which could be helpful to obtain a higher cell density with reduced byproduct formation. 

The G-XYL-opt strain was constructed by recombination with the plasmid of S17 containing the optimized promoter combination (pBB3cH-XYL-opt) to exclude the background effect.

**Figure 3:**
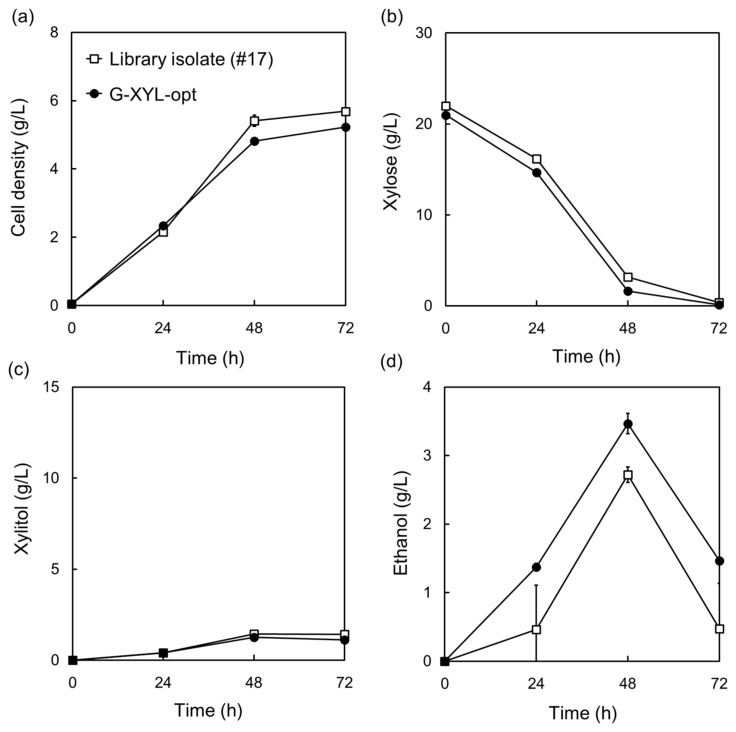
Confirmation of the optimized promoters for a heterologous xylose-metabolic pathway in *Komagataella phaffii*. The optimized promoters identified from a best-growing library isolate (#17) were identified, reconstructed, and introduced into wild-type *K. phaffii*, resulting in the G-XYL-opt strain. (**a**) Cell density, (**b**) xylose consumption, (**c**) xylitol production, and (**d**) ethanol production in YP medium containing 20 g/L xylose and 200 μg/mL hygromycin B at 30 °C and 130 rpm. Data represent the mean ± standard deviation of three independent biological replicate, with error bars reflecting the observed variability.

However, both strains showed similar xylose metabolism and growth profiles for 72 h, confirming that the strain with the optimal promoter-gene combination demonstrated excellent xylose availability without additional mutation (Figure 3).

### 2.3. Cellulosic Hydrolysate Fermentation

The GS115 strain, the parental strain of the G-XYL-opt strain, is a *his4-* auxotroph that cannot synthesize histidine [43]. Therefore, it was anticipated to exhibit growth defects in cellulosic hydrolysate lacking an organic nitrogen source. For cellulosic hydrolysate fermentation, the strain background was changed to the prototrophic X-33 strain by expressing the pBB3cH_XYL-opt, resulting in the X-XYL-opt strain.

**Figure 4:**
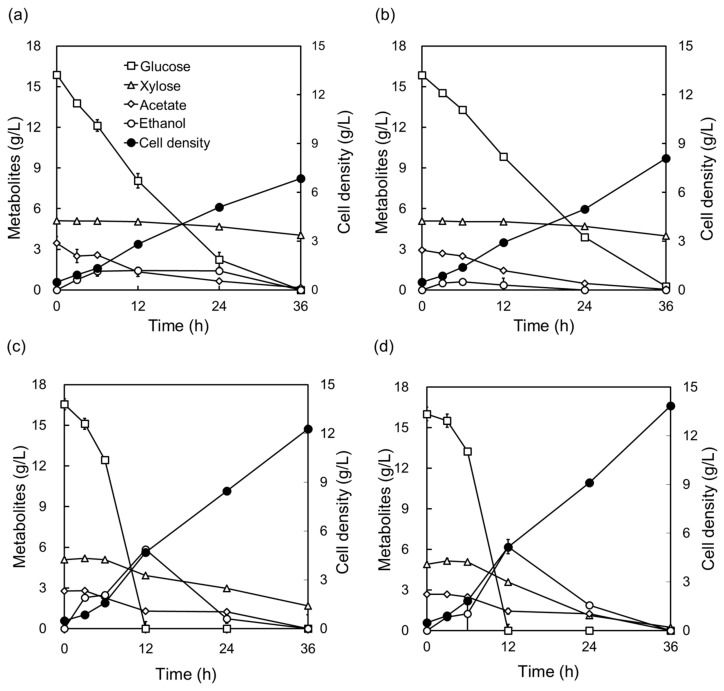
Engineered *Komagataella phaffii* for the utilization of cellulosic biomass hydrolysates. Prototrophic *K. phaffii* X-33 strain was transformed with an empty vector (X-control: (**a**,**c**)) and the optimized xylose pathway (X-XYL-opt: (**b**,**d**)), respectively. Fermentations were performed in 10% (*w*/*w*) kenaf hydrolysates under two conditions: (**a**,**b**) without any organic nitrogen supplementation, and (**c**,**d**) supplemented with YP medium (1% yeast extract, 2% peptone) and 200 μg/mL hygromycin B. All fermentations were conducted at 30 °C and 130 rpm. Data represent the mean ± standard deviation of three independent biological replicate, with error bars reflecting the observed variability.

Acid pretreatment, followed by enzyme saccharification of 15% (*w*/*w*) dried kenaf powder, yielded 26 g/L glucose and 8.5 g/L xylose. Compared to previous studies, these yields are higher than those obtained from acid hydrolysis alone (24.3 g/L glucose and 13.2g/L xylose) but lower than those achieved with alkaline deacetylation, followed by acid hydrolysis (28.4 g/L glucose and 17.3 g/L xylose) [11]. These differences highlight the impact of pretreatment method on sugar release efficiency [44]. The engineered strain *K. phaffii* X-XYL-opt demonstrated the ability to grow in kenaf cellulosic hydrolysate without any supplementation of organic nitrogen sources, achieving a cell mass of 8.7 g DCW/L medium (Figure 4b). This growth suggests that X-XYL-opt can synthesize the essential amino acid required for growth directly from the components present in cellulosic hydrolysate.

YP addition was used to add soluble protein and amino acids that are lacking in biomass fermentation [45,46]. When a nitrogen source was supplied, the cell mass increased proportionally to the concentration of added YP. Specifically, with YP10 (1% yeast extract and 2% peptone), X-XYL-opt grew to a cell mass of 13.9 g DCW/L at 36 h, at which X-XYL-opt exhibited a higher maximum growth rate than the control strain (X-control) (Figure 4). In addition, xylose consumption and growth rate were improved by utilizing the X-XYL-opt compared to the control strain. The 36-h fermentation indicates that the control strain consumed only 60-66% of the xylose in kenaf hydrolysate (Figure 4c). In comparison, the xylose-metabolizing strains consumed more than 95% (Figure 4d), and cell mass was from 10 to 13% higher than that of the control.

We developed a genetically engineered strain capable of utilizing xylose as a substrate and observed improvements in xylose metabolism and cell growth compared to the control strain. Although *Komagataella phaffii* is generally known to be unable to metabolize xylose, we confirmed that the X-control consumed some xylose during fermentation with cellulosic biomass hydrolysate (Figure 4c). These results may be explained by findings showing that xylose metabolism is possible even in non-engineered stains when methanol is supplied (Supplementary Figure S3). Xylose metabolism in native *Komagataella phaffii* is thought to result from the activation of metabolic pathways for utilizing alternative carbon sources when methanol supplied under glucose-depressed conditions. However, the X-XYL-opt strain developed in this study demonstrates the ability to metabolize xylose as a sole carbon source, without the need for methanol supplementation. This methanol-free xylose metabolism offers significant industrial advantages, particularly in the context of sustainable production using cellulosic biomass.

## 3. Discussion

Xylose-metabolizing yeasts have been constructed by introducing heterologous pathways based on (1) xylose oxidoreductase or (2) xylose isomerase [47]. In both pathways, xylulose serves as the intermediate, converted into xylulose-5 phosphate and further to ethanol via the pentose phosphate pathway. In several studies, the xylose oxidoreductase pathway exhibited faster xylose consumption than the xylose isomerase pathway in engineered yeasts [47,48].

In exclusive xylose conditions, ethanol, acting as an intermediate metabolite, serves as a product to validate the xylose metabolic process, undergoes temporary production, functions as a carbon source for growth, and is no longer present once fermentation is complete.

To improve xylose metabolism in the cofactor-dependent oxidoreductase pathway, optimization of the expression levels of enzymes involved in the xylose metabolism pathway is necessary [48]. Previous studies have also demonstrated that the activity ratio of XR and XDH exerts an essential effect on xylose metabolism, ethanol production, and xylitol accumulation. For instance, regulating gene expression by constructing the *S. cerevisiae* library with varying copy number ratios of XR/XDH/XK, followed by the selecting the best strain on xylose medium, resulted in more efficient xylose utilization with a lower activity ratio of XR and XDH, yielding 2.34-fold-higher xylose consumption rate than the parental strain [4]. These results align with our findings and are also consistent with a dynamic simulation study using *S. cerevisiae*, where a combination ratio for *XYL1:XYL2:XYL3* of 1:≥10:≥4 led to minimal xylitol formation [49]. 

A high concentration of xylose confers a selective pressure to strains. In the presence of a plasmid construct optimized for xylose metabolism, the enrichment medium’s metabolism and growth would be faster than that with less beneficial strains, providing a numerical advantage in the culture environment [50]. Through the enrichment process for the promoter library, it is possible to select efficient strains with the ideal promoter combination in a simple approach, without constructing all plasmids. However, several rounds of enrichment under high-concentration xylose conditions could result in adaptive evolution for strains, inducing genetic mutations in the introduced plasmid and genes, thereby affecting xylose metabolism (i.e., background effect) [26,32,33]. The background effect possibly arising from genetic mutations could lead to false positives. Since the selection of individual strains after xylose enrichment was based on cell growth rate, some mutations may have occurred in a direction favorable to cell growth rather than xylose metabolic rate. Additionally, the promoter activity may vary depending on the culture condition (i.e., carbon substrate) [51]. 

Engineered xylose-metabolizing strains exhibit accelerated growth when utilizing kenaf hydrolysates containing xylose, with the magnitude of the growth rate difference increasing proportionally to the xylose content in the biomass hydrolysates [52]. Although *K. phaffii* is a Crabtree-negative strain (different from the Crabtree-positive *S. cerevisiae* strain) that does not produce ethanol under aerobic and glucose-excess conditions [53], our result showed evident ethanol production by *K. phaffii* X-XYL-opt even under oxygen-rich conditions, aligning with findings from previous studies [54,55,56]. When cells are grown to a high density, respiratory metabolism can convert into respiratory-fermentation metabolism [57]. This phenomenon might have enabled *K. phaffii* to produce ethanol using cellulosic biomass as a substrate [58].

*K. phaffii* demonstrates a higher expression of mitochondrial genes involved in respiration compared to *S. cerevisiae* [59]. Consequently, cellular energy metabolism in *K. phaffii* in our study may have been more directed toward the respiration pathway than alcoholic fermentation, leading to improved cellular growth but reduced ethanol production. From an industrial perspective, the fermentation time and cell mass yield are directly correlated with process costs. The *K. phaffii* X-XYL-opt developed in this study exhibits both a high growth rate and high cell density, along with the ability to efficiently utilize cellulosic biomass through complete xylose conversion, enhancing its potential for facilitating viable bioprocesses.

## 4. Materials and Methods

### 4.1. Plasmids and Strains

All strains and plasmids used in this study are listed in Table 1. The *K. phaffii* GS115 and X-33 strains were purchased from Invitrogen (Waltham, MA, USA). The pBB3cH_Cas9 plasmid [60] was purchased from Addgene (Watertown, MA, USA; plasmid #104912).

### 4.2. Culture Conditions

Yeast strains were grown at 30 °C, with shaking at 200 rpm, in YP medium (10 g/L yeast extract and 20 g/L peptone) containing 20 g/L glucose. Hygromycin B (200 μg/mL) was added to the medium when required. For xylose fermentation, the yeast was cultured at 30 °C and 130 rpm, with an initial cell density of 0.05 g/L, in a 100 mL Erlenmeyer flask containing 20 mL YP medium supplemented with 20 g/L xylose and, when applicable, 200 μg/mL hygromycin.

### 4.3. Genome Integration of a Heterologous Xylose-Metabolic Pathway

The *K. phaffii* G-XYL-strong strain was constructed by genome integration of a heterologous xylose-metabolic pathway by Cas9. The gRNA sequence (GCGGCAGTAATTGATATCGTAGG) targeting the upstream of the *FLD1* gene [61] was used to modify the pBB3cH-Cas9 plasmid [62] into the pBB3cH-Cas9-FLD1UP plasmid using the primers in Table S2, based on the fast-cloning method [63]. For Donor DNA preparation (*GAPDHp_XYL1_ICL1t_ENO1p_XYL2_DAS1t_PET9p_XYL3_CYC1t*), 9 DNA fragments were generated by the primers in Table S2 and linked by cas9-assisted in vivo assembly, as described previously [64]. The pBB3cH-Cas9-FLD1UP plasmid (1 μg) and the donor DNA (1 μg) were introduced into the *K. phaffii* GS115 strain by electroporation (2000 mV, 25 μF, and 200 Ω) using an electroporator (ECM 630, Harvard Bioscience Inc., Holliston, MA, USA), as described previously [62].

### 4.4. Construction of Promoter Library for Xylose-Metabolic Pathway

To construct the promoter library, we utilized three constitutive promoters with varying strengths (strong, medium, and weak), paired with each of the xylose-metabolic gene (*XYL1*, *XYL2*, and *XYL3*), resulting in 27 possible combinations (Figure 2a). The choice of promoters and their design were based on previously established methods for identifying and characterizing functional promoters in *Komagataella phaffii* [65]. The primers listed in Table S3 were designed to enable high-throughput DNA assembly, ensuring compatibility with the NEBuilder^®^ HiFi DNA Assembly Master Mix (New England Biolabs Inc., Ipswich, MA, USA) protocol. The sequences included appropriate overlaps (20–30 bp) to facilitate seamless ligation during the assembly process. 

The ligated vectors were transformed into *E. coli* and cultured on LB agar plates containing 50 µg/mL hygromycin B at 37 °C. To confirm the diversity of the transformants, 8 colonies were randomly selected and analyzed via colony (Supplementary Table S4). The transformants (10^4^ cells) were collected, and the plasmids were extracted using the Exprep™ Plasmid SV (GeneAll Biotechnology Co., Seoul, Republic of Korea). The promoter library plasmids (pBB3cH-Library) were then introduced into *K. phaffii* GS115, and the resulting transformants were collected as the yeast library (10^4^ cells).

Enrichment of the yeast library was performed in YP medium containing 200 g/L of xylose and 200 μg/mL of hygromycin B at 30 °C and 130 rpm. Cells were sub-cultured into fresh medium every 4 days, with an initial cell density of 0.05 g/L. After enrichment, 4 single colonies were isolated and evaluated in YP medium containing 20 g/L xylose and 200 μg/mL hygromycin B. The colonies were also subjected to colony PCR, using the primers in Table 1, to confirm the promoters of each gene.

### 4.5. Cellulosic Biomass Hydrolysate Fermentation

Kenaf biomass was harvested in 2019 from Hwaseong, Gyeongi-do, Republic of Korea. The biomass was dried at 60 °C for 24 h, milled by a grinder, and stored at −80 °C until use [11]. For pretreatment, 1% (*w*/*v*) H_2_SO_4_ was added to 15% (*w*/*w*) kenaf powder. The mixture was autoclaved at 121 °C for 30 min and neutralized to pH 6.0 using 10 N NaOH. Enzymatic hydrolysis was performed by adding Cellic CTec2 (Novozymes, Bagsværd, Denmark) to the pretreated biomass. The hydrolysis reaction was conducted at 30 °C and 130 rpm for 72 h, followed by centrifugation at 3134× *g* for 5 min. The supernatant was supplemented with YP medium (1% yeast extract, 2% peptone) and 200 μg/mL hygromycin B for fermentation, with an initial cell density of 0.5 g/L.

### 4.6. High-Performance Liquid Chromatography

Cell cultures were diluted 10-fold and centrifuged at 15,928× *g* for 10 min, and the resulting supernatant was collected for analysis. For kenaf hydrolysate fermentation, the supernatants were additionally filtered using a 0.2 μm syringe filter prior to analysis. An HPLC system (Agilent 1260, Agilent Technologies, Santa Clara, CA, USA) equipped with a Rezex-ROA Organic Acid H^+^ column (8%, 150 mm × 4.6 mm; Phenomenex Inc., Torrance, CA, USA) was used. The analysis was performed at 50 °C, with 0.005 N H_2_SO_4_ as the mobile phase, at a flow rate of 0.6 mL/min [66].

### 4.7. Statistical Analysis

Fermentation data were analyzed using Student’s *t*-test or one-way ANOVA with the IBM SPSS software package (version 27, Armonk, NY, USA). Duncan’s multiple range test was used to compare means, and differences were considered significant at *p* < 0.05. Each experiment was carried out in triplicate.

## Figures and Tables

**Table 1:** Strains and plasmids used in this study.

Strains and Plasmids	Features ^1^	Reference
GS115	*Komagataella phaffii his4*	Invitrogen^TM^
G-XYL-strong	GS115 *GAPDHp_XYL1_ICL1t_ENO1p_XYL2_DAS1t_PET9p_XYL3_CYC1t*	This study
G-control	GS115 pBB3cH	This study
G-XYL-opt	GS115 pBB3cH-XYL-opt	This study
X-33	*Komagataella phaffii*, prototrophic	Invitrogen^TM^
X-control	X-33 pBB3cH	This study
X-XYL-opt	X-33 pBB3cH-XYL-opt	This study
pBB3cH	Low-copy plasmid with CEN6/ARS4 and *hph* (constructed by removing Cas9 from pBB3cH-Cas9)	This study
pBB3cH-Cas9	pBB3cH harboring Cas9 and sgRNA	[49]
pBB3cH-Cas9-FLD1UP	pBB3cH-Cas9 with sgRNA targeting the upstream of the *FLD1* gene	This study
pBB3cH-Library	pBB3cH harboring a xylose pathway with promoter library; *promoter1-XYL1-ICL1t-promoter2-XYL2-DAS1t-promoter3-XYL3-CYC1t*	This study
pBB3cH-XYL-opt	pBB3cH-*RSP2p-XYL1-ICL1t-ENO1p-XYL2-DAS1t-TKL1p-XYL3-CYC1t*	This study

^1^ The *XYL1*, *XYL2*, and *XYL3* genes are derived from *Scheffersomyces stipitis*.

## Data Availability

The original contributions presented in the study are included in the article/Supplementary Materials; further inquiries can be directed to the corresponding authors.

## Supplementary Materials

The following supporting information can be downloaded at https://www.mdpi.com/article/10.3390/molecules29235695/s1, Figure S1: Expression levels of the *XYL1* gene under the control of *Komagataella phaffii GAPDH* promoter and *Saccharomyces cerevisiae TDH3* promoter; Figure S2: Xylose fermentation profiles at 48 h by *Komagataella phaffii* strains selected through high concentration of xylose enrichment process; Table S1: Identification of combinatorial promoters for *XYL1*, *XYL2*, and *XYL3* in xylose-enriched *Komagataella phaffii* isolates; Figure S3: Comparison of cell growth and xylose metabolism in engineered *Komagataella phaffii*; Table S2: Primers used in this study; Table S3: Primers used to construct promotor library plasmid; Table S4: Promoter distribution of 8 randomly selected *E. coli* transformants of the promoter library.
