# Supplementary material for: Metabolic Engineering of Komagataella phaffii for Xylose Utilization from Cellulosic Biomass
Source: Molecules. 2024 Dec 2;29(23):5695. doi: 10.3390/molecules29235695 (PMC11643697; doi:10.3390/molecules29235695)
Supplement: Supplementary file 1 [file molecules-29-05695-s001.zip › molecules-3319275-supplementary.pdf]

Supplementary materials for

Metabolic Engineering of *Komagataella phaffii* for Xylose Utilization from Cellulosic Biomass

Jongbeom Park [1], Sujeong Park [1], Grace Evelina [1], Sunghee Kim [2], Yong-Su Jin [3], Won-Jae Chi [4], In Jung Kim [5,*] and Soo Rin Kim [1,2,*]

[1] School of Food Science and Biotechnology, Kyungpook National University, Daegu 41566, Republic of Korea; bum5743@gmail.com (J.P.); tya198@naver.com (S.P.); graceevelina@gmail.com (G.E.)
[2] Research Institute of Tailored Food Technology, Kyungpook National University, Daegu 41566, Republic of Korea; sunghee.kimmmm@gmail.com
[3] Department of Food Science and Human Nutrition, University of Illinois at Urbana-Champaign,
Urbana, IL 61801, USA; ysjin@illinois.edu
[4] Species Diversity Research Division, National Institute of Biological Resources, Incheon 22689, Republic of Korea; wjchi76@korea.kr
[5] Department of Food Science & Technology, Institute of Agriculture and Life Science, Gyeongsang National University, Jinju 52825, Republic of Korea
* Correspondence: ij0308@gnu.ac.kr (I.J.K.); soorinkim@knu.ac.kr (S.R.K.)

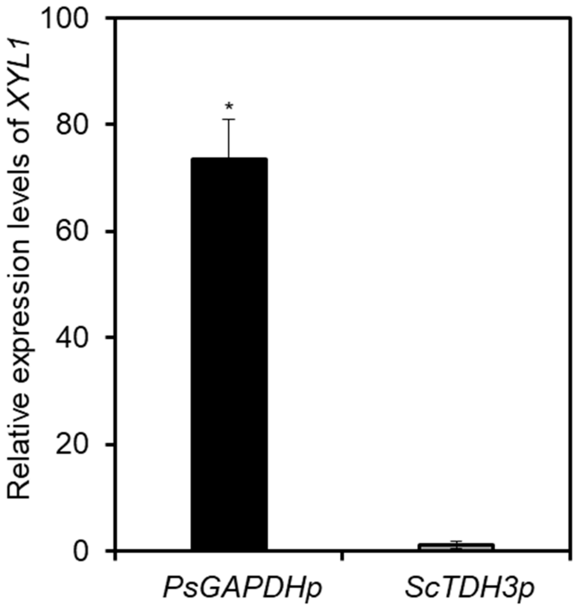

Figure S1. Expression levels of the *XYL1* gene under the control of *Komagataella phaffii GAPDH* promoter and *Saccharomyces cerevisiae TDH3* promoter. The *RSC1* gene was used as a reference gene. Data are represented as mean ± standard deviation based on three biological replicates. Statistical significance between the expression levels under different promoters was determined using Student's t-test ($P < 0.05$).

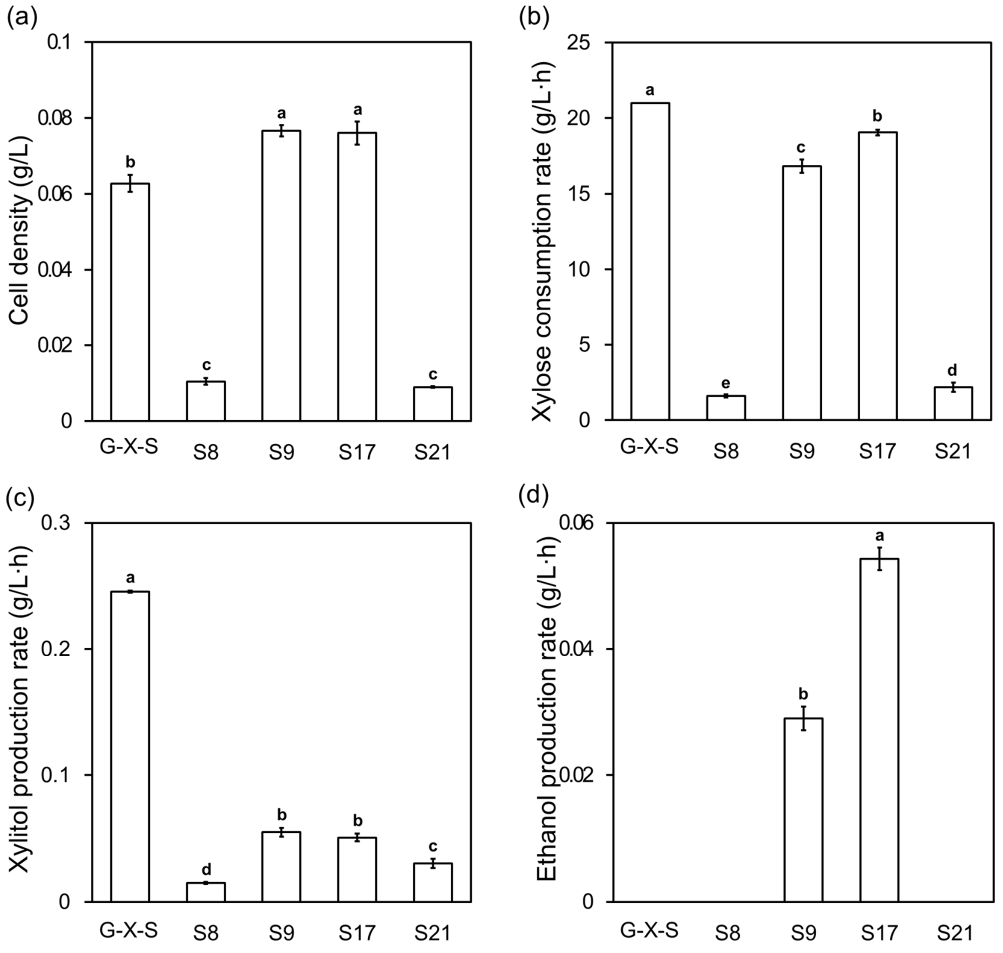

Figure S2. Xylose fermentation profiles at 48 h by *Komagataella phaffii* strains selected through high concentration of xylose enrichment process. All strains were cultured in YP medium containing 20 g/L xylose and 200 μg/mL hygromycin B. (**a**) Growth profile, (**b**) x ylose consumption rate, (**c**) xylitol production rate, and (**d**) ethanol production rate. T he strains include G-X-S (G-XYL-strong strain) and isolated strains(S8, S9, S17, and S21) with enhanced xylose utilization capabilities from the enrichment process. Data are presented as mean ± standard deviation based on three biological replicates. Different letters above the bars indicate statistically significant differences between the strains for each parameter.

Table S1. Identification of combinatorial promoters for *XYL1*, *XYL2*, and *XYL3* in xylose-enriched *Komagataella phaffii* isolates.

Isolates	Promoter region 1[1]	Promoter region 2	Promoter region 3
S8	*RSP2p*	*TPI1p*	*GPM1p*
S9	*RSP2p*	*ENO1p*	*TKL1p*
S17	*RSP2p*	*ENO1p*	*TKL1p*
S21	*PGK1p*	-[2]	*GPM1p*

[1] Promoters 1, 2, and 3 represent promoters regulating gene expression of *XYL1*, *XYL2*, and *XYL3*, respectively.

[2] not identified

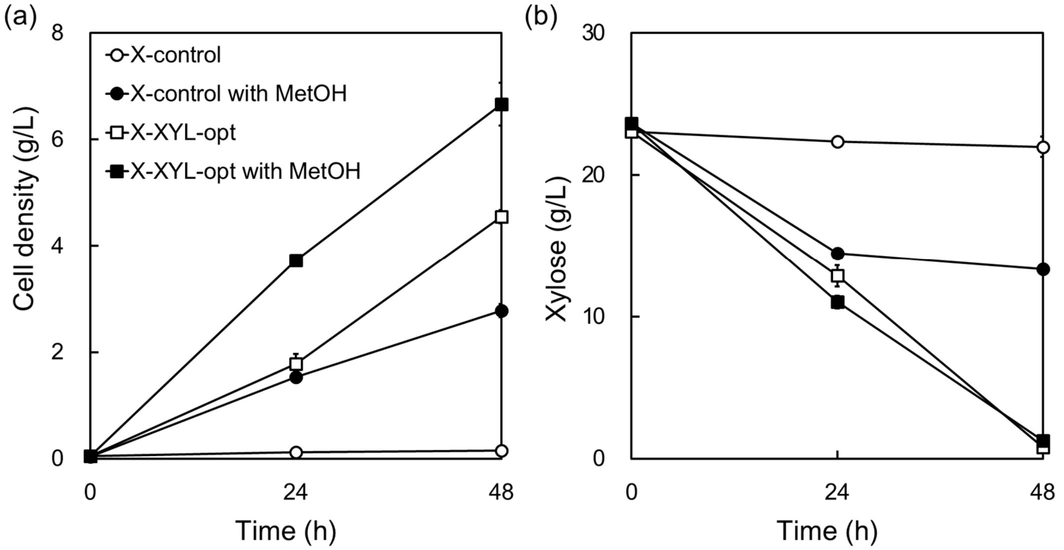

Figure S3. Comparison of cell growth and xylose metabolism in engineered *Komagataella phaffii*. The Fermentation of X-control and X-XYL-opt strains was compared with and without 1% methanol (MetOH), showing (a) cell density and (b) xylose consumption in YP medium containing 20 g/L xylose supplemented with 200 μg/mL hygromycin B at 30°C and 130 rpm. Data represent the mean ± standard deviation of three independent biological replicate, with error bars reflecting the observed variability.

Table S2. Primers used in this study

Primers	Sequences (5'-)	Description
kim1159	ACGAAACGAGTAAGCTCGTCGCGGCAGTAATTGATATCGTGTTTTAGAGCTAGAAATAGCAAG	FC_FLD1UP_F
kim1160	AGCTTACTCGTTTCGTCCTCACGGACTCATCAGGCGGCACATGGTGTTTTGATAGTTGT	FC_FLD1UP_R
kim1234	GAGAAGTTTTTTTACCCCTCTCCACAGATC CTACAATTGGAGCCCAATC	Donor_FLD1UP_UP_F1
kim1231	GATCAATGGAAAGGAAGTTC	Donor_FLD1UP_UP_R1
kim1276	ATTATCAGACGCTATTGCAAGAACTTCCTTTCCATTGATC AGGATCCTTTTTTGTAGAAATG	Donor_GAPDHp_F
kim1277	GTAACCAGAGTTCAACTTAATAGAAGGCAT TGTGTTTTGATAGTTGTTCAATTG	Donor_GAPDHp_R
kim1278	TTCAATCAATTGAACAACTATCAAAACACA ATGCCTTCTATTAAGTTGAAC	Donor_XYL1_F
kim1279	TTATCTTAACTACTTAGGAACTCCTATTAC TTAGACGAAGATAGGAATCTTG	Donor_XYL1_R
kim1280	GACTGGGACAAGATTCCTATCTTCGTCTAA GTAATAGGAGTTCCTAAGTAG	Donor_ICL1t_F
Kim1343	AGAAAAAGATTTCTTTCAAGAAG	Donor_ICL1t_R
Kim1422	TCCTTCTTGAAAGAAATCTTTTTCT ATGAAAGAGTGAGAGGAAAG	Donor_ENO1p_F1
Kim1423	ACACCAAGGAAGGGTTAGCAGTCAT TTTTAGATGTAGATTGTTATAATTGTG	Donor_ENO1p_R1
Kim1402	ATGACTGCTAACCCTTCC	Donor_XYL2_F1
Kim1403	AACTAAAACTGTAAAGACTTCCCGT TTACTCAGGGCCGTCAATG	Donor_XYL2_R1
Kim1404	GTGTCTCATTGACGGCCCTGAGTAA ACGGGAAGTCTTTACAGTTTTAG	Donor_DAS1t_F1
Kim1405	CCAAATTGTAATCATCAGTG	Donor_DAS1t_R1
Kim1428	ATAATCACTGATGATTACAATTTGG TAGAAAATTCACCACTGTC	Donor_PET9p_F1
Kim1429	GAGCATCAAATGGGGTAGTGGTCAT TTTGGAATATTATAGATTTGTAAGAAAG	Donor_PET9p_R1
Kim1458	ATGACCACTACCCCATTTG	Donor_XYL3_F1
Kim1459	AAGCGTGACATAACTAATTACATGA TTAGTGTTTCAATTCACTTTCC	Donor_XYL3_R1
Kim1457	TCATGTAATTAGTTATGTCACG	Donor_CYC1t_F1
Kim1558	CAGAGATTCCTGCAGAATGGGACACAAGCGCCAGCGTCTTT GCAAATTAAAGCCTTCGAG	Donor_CYC1t_R1
kim1232	AAAGACGCTGGCGCTTG	Donor_FLD1UP_DN_F1
kim1235	CCGGGTAGATTTTTCCGTAACCTTGGTGTC TCGACAACACCAGCACC	Donor_FLD1UP_DN_R1
Kim1469	TAGGTCGTTCGCTCCAAG	Confirm_XYL1_F1

Kim1470	TTCGTAGTCGAAGTTGTCACC	Confirm_XYL1_R1
Kim1486	ATGAAAGAGTGAGAGGAAAGTACC	Confirm_ENO1p_F1
Kim1488	TCTTGGACAACAATTCGGAAC	Confirm_TPI1p_F1
Kim1489	TTTGGGTATTGGAATGTTGGTAGC	Confirm_THI11p_F1
Kim1487	AAACGAAGTTACCGATTCTACC	Confirm_XYL2_R1
Kim1490	AGTTGTCTACTTCCGTCG	Confirm_PET9p_F1
Kim1492	GTATTGGCAGGCTGATTTC	Confirm_GPM1p_F1
Kim1493	ACCTTTGTCCCACTAACC	Confirm_TKL1p_F1
Kim1491	AAACATGGTCAAGGGCATC	Confirm_XYL3_R1

Table S3. Primers used to construct promoter library plasmid

Primer	Sequence (5'-)	Description
FC_del_Cas9_F	CTTTCCAGATCTGATCACTTGTGAAGACGACCATCGTCTCACCATGGTCTCACCATTCA TGTAATTAGTTATGTCACG	Fastcloning for Cas9 deletion of pBB3cH
FC_del_Cas9_R	GATCAGATCTGGAAAGCGAAGACGACTCCCGTCTCGCTCCGGTCTCGCTCCATAGTTG TTCAATTGATTGAAATAGG	
HF_BB3cH_F	ATGACCACTACCCCATTTG	Construction of pBB3cH_RAXYL through NeBuilder ® Hifi DNA assembly
HF_BB3cH_R	AGATCAAAGGATCTTCTTGAG	
HF_GAPDHp_F	GGATCTCAAGAAGATCCTTTGATCTAGGATCCTTTTTTGTAGAAATG	Promoter fragment of GAPDH, PGK1, and RSP2 for XYL1
HF_GAPDHp_R	CAGAGTTCAACTTAATAGAAGGCATTGTGTTTTGATAGTTGTTCAATTG	
HF_PGK1p_F	GGATCTCAAGAAGATCCTTTGATCTAAGTTGGTACCCAGCCG	
HF_PGK1p_R	CAGAGTTCAACTTAATAGAAGGCATTTTCGTAATCAATTGGGCTATGC	
HF_RSP2p_F	GGATCTCAAGAAGATCCTTTGATCTAATATCGGGGATTGGTTG	
HF_RSP2p_R	CAGAGTTCAACTTAATAGAAGGCATATTAACTACACTGAAAAAGTCG	
HF_XYL1_F	ATGCCTTCTATTAAGTTGAAC	XYL1 genes fragment
HF_XYL1_R	TTAACTACTTAGGAACTCCTATTACTTAGACGAAGATAGGAATCTTG	
HF_ICL1t_F	GGACAAGATTCCTATCTTCGTCTAA GTAATAGGAGTTCCTAAGTAG	ICL1 terminator fragment for XYL1
HF_ICL1t_R	AGAAAAAGATTTCTTTCAAGAAG	
HF_ENO1p_F	TCCTTCTTGAAAGAAATCTTTTTCTATGAAAGAGTGAGAGGAAAG	Promoter fragment of ENO1, TPI1, and THI11 for XYL2
HF_ENO1p_R	ACACCAAGGAAGGGTTAGCAGTCATTTTAGATGTAGATTGTTATAATTGTG	
HF_TPI1p_F	TCCTTCTTGAAAGAAATCTTTTTCTTTCAACGAGACACTCTTC	

HF_TPI1p_R	ACACCAAGGAAGGGTTAGCAGTCAT TGTGTTTGTGATAGATCTTG	
HF_THI11p_F	TCCTTCTTGAAAGAAATCTTTTTCT CAATAGGAGCACCTATAGTT	
HF_THI11p_R	ACACCAAGGAAGGGTTAGCAGTCATGATGATTTATTGAAGTTTCCAAAG	
HF_XYL2_F	ATGACTGCTAACCCTTCC	XYL2 gene fragment
HF_XYL2_R	AACTAAAACTGTAAAGACTTCCCGT TTACTCAGGGCCGTCAATG	
HF_DAS1t_F	GTGTCTCATTGACGGCCCTGAGTAAACGGGAAGTCTTTACAGTTTTAG	DAS1 terminator fragment for XYL2
HF_DAS1t_R	CCAAATTGTAATCATCAGTG	
HF_PET9p_F	ATAATCACTGATGATTACAATTTGGTAGAAAATTCACCACTGTC	Promoter fragment of PET9, GPM1, and TKL1 for XYL3
HF_PET9p_R	GAGCATCAAATGGGGTAGTGGTCATTTTGGAATATTATAGATTTGTAAGAAAG	
HF_GPM1p_F	ATAATCACTGATGATTACAATTTGG TTGATCCAGTGTTAGATCC	
HF_GPM1p_R	GAGCATCAAATGGGGTAGTGGTCATTGTTTGTTTGTGTAATTGAAAG	
HF_TKL1p_F	ATAATCACTGATGATTACAATTTGGACCCGTAATAATTGCC	
HF_TKL1p_R	GAGCATCAAATGGGGTAGTGGTCATTGTGTAGAGTGGATGTAG	
HF_XYL3_F	ATGACCACTACCCCATTTG	XYL3 gene fragment
HF_XYL3_R	AAGCGTGACATAACTAATTACATGA TTAGTGTTTCAATTCACTTTCC	
HF_CYC1t_F	TCATGTAATTAGTTATGTCACG	CYC1 terminator fragment for XYL3
HF_CYC1t_R	CAGAGATTCCTGCAGAATGGGACACAAGCGCCAGCGTCTTT GCAAATTAAAGCCTTCGAG	

Table S4. Promoter distribution of 8 randomly selected *E. coli* transformants of the promoter library

Groups	Promoter	Number of colonies
Group 1	*GAPDHp*	5
	PGK1p	0
	RSP2p	1
	Not identified	2
Group 2	*ENO1p*	0
	TPI1p	0
	THI11p	5
	Not identified	3
Group 3	*PET9p*	1
	GPM1p	4
	TKL1p	0
	Not identified	3
